# COVID-19 in 2021

**DOI:** 10.3390/v13102098

**Published:** 2021-10-18

**Authors:** Kenneth Lundstrom, Alaa A. A. Aljabali

**Affiliations:** 1 PanTherapeutics, 1095 Lutry, Switzerland; 2 Department of Pharmaceutics and Pharmaceutical Technology, Faculty of Pharmacy, Yarmouk University, Irbid 21163, Jordan; alaaj@yu.edu.jo

## 1. Introduction

The Special Issue on Vaccines and Therapeutics against Coronaviruses, which was launched in early 2021, has attracted the scientific community at large, and more than 20 manuscripts have been accepted for publication. This is not surprising as a PubMed search for “COVID-19 therapeutics” and “COVID-19 vaccines” resulted in 39,249 and 11,005 hits, respectively. The types of articles include reviews, scientific articles, and brief reports. Interestingly, the spectrum of the subjects ranges from basic research to clinical execution representing the whole repertoire of translational medicine. The topics cover both vaccines and therapeutics. Several publications describe the evaluation of repurposed and novel drugs and natural compounds for the treatment of COVID-19 and screening of drugs against COVID-19. Various aspects of the development of vaccines against SARS-CoV-2 are described in four reviews dealing with vaccine research approaches, including vaccine design, an overview of clinical trials, and future aspects of vaccine development. In the context of the future handling of the pandemic, the effect of mutations in the SARS-CoV-2 genome and particularly in the spike (S) protein are discussed. From a clinical point of view, pharmacogenetics approaches to providing improvement in COVID-19 treatment are presented. Moreover, long-COVID and post-COVID health complications and the molecular mechanisms behind these issues are described. The anti-tumor response by SARS-CoV-2 in isolated cases of lymphomas provides an interesting aspect to COVID-19. On a more fundamental research level, the interaction of the receptor-binding domain (RBD) of the SARS-CoV-2 S protein with the angiotensin-converting enzyme 2 (ACE2) host cell receptor is presented.

## 2. Therapeutic Approaches to COVID-19

To address the unmet needs for COVID-19 therapy, screening 5406 compounds, including FDA-approved drugs and bioactive substances with activity against MERS-CoV, identified several cardiotonic agents such as atovaquone, an anti-malarial drug, and ciclesonide, an inhalable corticosteroid [1]. Using SARS-CoV-2 for testing combination therapy with remdesivir in Vero-E6 and Calu-3 cells, circlesonide, nelfinavir, and camostat were identified as potential therapeutic options against MERS-CoV, which also can provide the basis for COVID-19 therapeutics. In another approach, high throughput screening of SARS-CoV-2 3C-like protease (3CLpro) inhibitor was conducted based on the AlphaScreen technology [2]. Screening of 91 natural product compounds identified all-trans retinoic acid (ATRA), which inhibited SARS-CoV-2 replication in Vero E6 and Calu-3 cells. Furthermore, ATRA also inhibited recent variants of concern (VoC) such as alpha, beta, gamma, and delta variants. Moreover, antiviral activity against SARS-CoV-2 of traditional herbal medicines has been evaluated. In this context, turmeric root and its bioactive ingredient curcumin in the form of curcumin-containing supplement capsules and pure curcumin effectively neutralized SARS-CoV-2 and reduced SARS-CoV-2 RNA levels in Vero E6 and Calu-3 cells [3]. These findings need to be further confirmed in animal models and in clinical trials. In another similar approach, the antiviral activity of glycyrrhizin, present in licorice root, showed potent inhibition of SARS-CoV-2 replication in vitro [4]. It was demonstrated that glycyrrhizin inhibited the SARS-CoV-2 main protease (Mpro) by blocking viral replication. Therefore, consumption of licorice root tea of black licorice may benefit COVID-19 patients, which needs to be subjected to clinic evaluation. In the case of repurposed drugs, ketotifen, naproxen, and indomethacin were evaluated for their effect on SARS-CoV-2 replication, in combination or alone [5]. Ketotifen combined with either indomethacin or naproxen enhanced the inhibition of viral yield substantially and showed no cytotoxic effects in vitro, which should encourage evaluation in humans.In attempts to prevent SARS-CoV-2 binding to the ACE2 receptor on host cells, Bromelain and Acetylcysteine (BromAc) with demonstrated synergistic action against glycoproteins were tested on recombinant SARS-CoV-2 spike and envelope proteins [6]. BromAc treatment caused a concentration-dependent inactivation of SARS-CoV-2 in vitro, which needs to be verified in vivo.

In one Brief Report, amantadine, which was previously approved as an antiviral drug against Influenza A virus, was evaluated for SARS-CoV-2 inhibition in Vero E6 cells [7]. Although amantadine demonstrated inhibition of SARS-CoV-2 replication at IC_50_ concentrations between 83 and 119 μM, which is above therapeutic levels for systemic administration, topical or intranasal administration might be feasible for therapeutic utilization in humans. In the other Brief Report, Aprotinin was combined with Avifavir^®^ or hydroxychloroquine (HCQ) for the treatment of moderate COVID-19-related pneumonia as recommended by the Russian Ministry of Health [8]. The combination of Aprotinin with both HCQ and Avifavir^®^ completely prevented the need to transfer patients to the intensive care unit (ICU). However, the Aprotinin and Avifavir^®^ treatments were superior compared to Aprotinin and HCQ. In another study, 743 COVID-19 patients were pre-treated with HCQ in South Korea [9]. There was no indication of any positive effect of HCQ treatment to lower the risk of SARS-CoV-2 infection significantly.

Ben-Zuk and co-authors provide a review on the prophylactic treatment of COVID-19 [10]. Information is presented on several antiviral drugs, repurposed anti-parasitic drugs, and novel drug screening. Briefly, the importance of prophylactic use of vitamin and dietary supplements, antibody-based treatment, and innate immune response boost is mentioned. All avenues need to be explored, and cautious optimism is welcome but, for example, the declaration of extraordinary protection against and treatment of COVID-19 with ivermectin needs to be analyzed based on well-designed and representative clinical trials. The recent accusations of flawed ivermectin trials and withdrawal of a preprint publication highlight the challenges and do not advance research or clinical applications [11]. 

## 3. Vaccine Development against COVID-19

Related to vaccine development, four reviews on different aspects such as the current scenario and future perspectives [12], description of clinical trials, emergency use authorization, and overcoming hurdles and barriers to overcome the COVID-19 pandemic [13,14] are included. In another review, the various domains of the SARS-CoV-2 S protein and their functions are dissected, and new variants are described in the light of vaccine development [15]. One difficulty with reviews on COVID-19 vaccines is the unprecedented speed of development in the field. Once peer-reviewed and accepted for publication, the information is already outdated.

## 4. Structural Biology, Drug Discovery, and Mutations

Structural biology plays an important part in the dissection of virus–host cell interaction as described for the densely glycosylated RBD of the SARS-CoV-2 S protein and the ACE2 host cell receptor [16]. The simulation of RBD-ACE2 complexing with glycans identified key residues at the RBD–ACE2 interface. For instance, the N501Y mutation may change the interaction of the RBD with the ACE2, the glycan on Asn90 of ACE2 can affect the binding to the RBD, and the MAN9 glycans on ACE2 decreased the RBD-ACE2 affinity.

Structural biology has also supported drug discovery by identifying natural products acting as SARS-CoV-2 Mpro inhibitors [17]. Based on computational approaches, natural products from the coneflower *Echinacea augustifolia* were discovered as potent inhibitors of Mpro and could be attractive drug candidates against SARS-CoV-2.

In the context of SARS-CoV-2 mutations/variants, some of the VoC have been postulated to enhance virus transmissibility and host immune evasion [18]. Of much concern, some mutations might reduce the efficacy of vaccines and monoclonal antibodies against SARS-CoV-2. Based on available SARS-CoV-2 sequences, the global prevalence of so-called “adaptive mutations” and “mutations identified in prolonged infections” in the RBD of the S protein was identified. The N501Y was the dominant mutation, present in 48.38% of cases, but recently a massive increase in the L452R and T478K mutations (delta variant) has been seen. The collected data can support the development of better infection control policies.

Another study investigated the risk of emergence of mutations in individuals treated with the Bamlanivimab monoclonal antibody [19]. Six COVID-19 patients infected with the alpha variant were treated with Bamlanivimab. The E484K mutation was discovered in five of the six treated patients. Although the sample size is small, it indicated a risk of the emergence of mutants in patients treated with monoclonal antibodies.

## 5. Clinical Findings

Several publications deal directly with clinical aspects of COVID-19, however from different angles. In one study, the association between bitter taste receptor 38 (T2R38) phenotypes and the severity of COVID-19 was investigated [20]. The COVID-19 patients were divided into supertaster (184 individuals), taster (371 individuals), and non-taster (192 individuals) phenotypes. The duration of symptoms in individuals subjected to treatment with azithromycin, dexamethasone, and +/− HCQ was 5 days for supertasters, 8.1 days for tasters, and 16.2 days for non-tasters showing a significant correlation between the T2R38 phenotypic expression and symptom duration.

In another study, the variability in drug responses in COVID-19 patients was evaluated based on pharmacogenetics as a means of improving patient outcomes [21]. The pharmacogenetics for the treatment of COVID-19 with remdesivir, oseltamivir, lopinavir, ritonavir, azithromycin, chloroquine, HCQ, ivermectin, and dexamethasone was investigated. Such prospective studies could provide recommendations for personalized COVID-19 therapy. Moreover, pharmacogenetics should be included in the evaluation of drug responses to emerging SARS-CoV-2 variants.

An important issue is the description of long-term effects (long-COVID-19) and the adverse events occurring post-COVID, which seem to affect the immune, hematological, pulmonary, cardiovascular, skeletomuscular, and nervous systems and mental health [22]. The possible molecular mechanisms associated with disease symptoms and outcomes are discussed. The conclusion, however, is that the overall COVID-19 pathology is characterized by cytokine storm, which leads to endothelial inflammation, microvascular thrombosis, and multiple organ failure. Finally, individual cases of complete remission of classical Hodgkin lymphoma (cHL) and follicular lymphoma (FL) have been detected after SARS-CoV-2 infection [23]. These findings are not extremely surprising as several RNA viruses have demonstrated oncolytic activity and have been commonly used for cancer therapy [24]. However, here, the authors hypothesize on the mechanism of anti-tumor responses of SARS-CoV-2 based on the RBD of the S protein binding to extracellular domains of the cluster of differentiation (CD) molecules, binding to gamma-tubulin component 3 (GCP3), interaction of the SARS-CoV-2 M protein with tubulin gamma-1 chain (TUBG1), the interaction of SARS-CoV-2 M and ORF3a proteins with gamma-tubulin ring complex components, the interaction of the RBD of the S protein with PD-1 signaling and the tumor necrosis factor receptor type 1-associated DEATH domain (TRADD) protein interacting with Epstein–Barr virus LMP-1.

## 6. Conclusions

The Special Issue on Vaccines and Therapeutics against Coronaviruses managed to assemble a comprehensive collection of research and clinical applications, naturally triggered by, and focusing on the current COVID-19 pandemic. It is truly a powerful demonstration of translational virology covering various aspects from bench to bedside, and we sincerely hope that the readers have profited from the broad description of methods and technologies. Most importantly, we hope that the science presented here can contribute to conquering the pandemic and to being better prepared for future emerging viral outbreaks, to be able to put them to rest before they reach pandemic proportions.
